# Circulating SMRP and CA‐125 before and after pleurectomy decortication for pleural mesothelioma

**DOI:** 10.1111/1759-7714.15264

**Published:** 2024-04-16

**Authors:** Juuso Paajanen, Ahmed Sadek, William G. Richards, Yue Xie, Emanuele Mazzola, Kristina Sidopoulos, John Kuckelman, Ritu R. Gill, Raphael Bueno

**Affiliations:** ^1^ The Thoracic Surgery Oncology Laboratory and the International Mesothelioma Program (http://www.impmeso.org), Division of Thoracic Surgery and the Lung Center, Brigham, and Women's Hospital Harvard Medical School Boston Massachusetts USA; ^2^ Deparment of Data Science Dana‐Farber Cancer Institute Boston Massachusetts USA; ^3^ Department of Radiology Beth Israel Deaconess Medical Center and Harvard Medical School Boston Massachusetts USA

**Keywords:** biomarkers, CA‐125, pleural mesothelioma, pleurectomy decortication, SMRP

## Abstract

**Background:**

Tumor recurrence remains the main barrier to survival after surgery for pleural mesothelioma (PM). Soluble mesothelin‐related protein (SMRP) and cancer antigen 125 (CA‐125) are established blood‐based biomarkers for monitoring PM. We prospectively studied the utility of these biomarkers after pleurectomy decortication (PD).

**Methods:**

Patients who underwent PD and achieved complete macroscopic resection with available preoperative SMRP levels were included. Tumor marker levels were determined within 60 days of three timepoints: (1) preoperation, (2) post‐operation, and (3) recurrence.

**Results:**

Of 356 evaluable patients, 276 (78%) had recurrence by the end of follow‐up interval. Elevated preoperative SMRP levels were associated with epithelioid histology (*p* < 0.013), advanced TNM (*p* < 0.001) stage, and clinical stage (*p* < 0.001). Preoperative CA‐125 levels were not significantly associated with clinical covariates. Neither biomarker was associated with survival or disease‐free survival. With respect to nonpleural and nonlymphatic recurrences, mean SMRP levels were elevated in patients with pleural (*p* = 0.021) and lymph node (*p* = 0.042) recurrences. CA‐125 levels were significantly higher in patients with abdominal (*p* < 0.001) and lymph node (*p* = 0.004) recurrences. Among patients with all three timepoints available, we observed an average decrease in SMRP levels by 1.93 nmol/L (*p* < 0.001) postoperatively and again an average increase at recurrence by 0.79 nmol/L (*p* < 0.001). There were no significant changes in levels of CA‐125 across the study timepoints (*p* = 0.47).

**Conclusions:**

Longitudinal changes in SMRP levels corresponded with a radiographic presence of disease in a subset of patients. SMRP surveillance could aid in detection of local recurrences, whereas CA‐125 could be helpful in recognizing abdominal recurrences.

## Graphical abstract

SMRP and Ca‐125 were collected within 60 days of three timepoints: (1) preoperation, (2) postoperation, and (3) recurrence. Mean levels of circulating SMRP, but not CA‐125, decreased significantly after surgery and elevated again at time of recurrence. SMRP levels were elevated in patients with local recurrences, while CA‐125 levels were associated with abdominal recurrences.

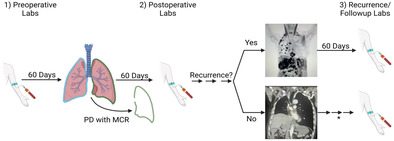

## INTRODUCTION

Pleural mesothelioma (PM) is an aggressive, asbestos caused cancer derived from the mesothelial cells of the pleura. The incidence of PM has slowly declined, while survival has remained unchanged with a median of 7 to 14 months in the US.^1^, ^2^ Surgery plays an important role in the diagnosis, staging, and management of PM. The primary goal of radical surgery is to remove all grossly visible, palpable, and viable tumors; that is, macroscopic complete resection (MCR).^3^ Tumor recurrence after surgery, which on average manifests within 11 to 14 months postoperatively, remains a significant barrier to long‐term survival.^4^, ^5^, ^6^ The current mainstay of postoperative surveillance in pleural mesothelioma is based on radiological imaging, which can be nonspecific due to postoperative changes and the unique morphology of the disease.

Circulating, blood‐based, biomarkers that accurately reflect disease burden could facilitate early diagnosis of recurrence during postoperative surveillance. Mesothelin is a glycoprotein expressed by normal mesothelial cells and found at high levels in many solid cancers, including PM.^7^ Serum soluble mesothelin‐related peptides (SMRP) have been suggested as a biomarker for PM as levels correlate with tumor size and disease progression.^8^ To date, serum or pleural SMRP is the only Food and Drug Administration (FDA)‐approved laboratory test for monitoring epithelioid and biphasic PM.^9^ Cancer antigen 125 (CA‐125) is a tumor antigen located in the cell surface of mesothelial cells.^10^, ^11^ Serum CA‐125 levels are most consistently elevated in epithelial ovarian cancer but can also be expressed by other malignancies and benign conditions.^12^, ^13^ Characteristics of circulating CA‐125 in PM patients have been less studied, but elevations have been reported in a subset of peritoneal mesothelioma patients.^14^, ^15^

Previous studies have focused on circulating biomarkers in advanced, unresectable PM.^8^, ^16^, ^17^, ^18^, ^19^ Our primary objective was to investigate the changes in circulating SMRP and CA‐125 in patients who underwent pleurectomy decortication (PD). In addition, we explored clinical factors associated with these markers before surgery and at the recurrence. Finally, we determined whether levels of SMRP or CA‐125 has a prognostic impact following MCR.

## METHODS

### Patients

We identified all PM patients (*N* = 555) who underwent PD between September 2009 and January 2022 from our institution's prospectively collected mesothelioma database. We excluded patients for whom MCR was not achieved, patients without preoperative SMRP available, and/or patients with any final pathology other than diffuse PM (i.e., localized mesothelioma or well‐differentiated papillary mesothelial tumor). Analyses of recurrence and disease monitoring for SMRP was limited to only patients with epithelioid and biphasic subtypes. This study was conducted in accordance with the amended Declaration of Helsinki. All participants gave written consent to IRB‐approved clinical trials (DFCI IRB 08–063) that allow for prospective clinical, treatment, and outcome analysis.

Prospectively gathered data was verified and augmented by chart review from electronic medical records (EMRs). Outcome and follow‐up data were obtained via chart review and directly contacting patients or referring doctors. Mortality data was obtained from the EMR, review of the Social Security Death Index or obituaries. Study follow‐up ended in January 2023.

Our institution's standardized preoperative work‐up and surgical approach has recently been reviewed elsewhere.^20^ Operative details were gathered directly from surgeon surveys or from operation notes. Clinical and pathological data collected included age, sex, histological subtype, extent of the resection, and tumor laterality. Data on induction, intraoperative, and adjuvant therapy were extracted from EMRs. The pathological stage was determined using the eighth edition of the tumor, node, and metastasis (TNM) AJCC classification.^21^

### Radiological assessment

Analysis of computed tomography (CT), positron emission tomography (PET)/CT, or magnetic resonance imaging (MRI) scans were performed by a dedicated thoracic radiologist (RRG). Pretreatment tumor volume (TV) was determined from CT scans as previously described.^22^ Clinical AJCC TNM stage was assessed according to the eighth edition. Quantitative clinical stage, which combines pretreatment TV with maximal fissural thickness (Fmax), was defined and categorized as described by Gill et al.^23^ Serial CT and PET/CT scans were evaluated to identify and categorize recurrences.

Tumor recurrence was defined as: (1) a pathology‐proven lesion, or (2) a new suspicious imaging (increasing in size or fluorodeoxyglucose F‐18 [FDG] avid). Anatomical location of the first recurrence was radiologically determined. Local recurrence was defined as a lesion at the ipsilateral hemithorax (IHT), including the neopleural space, diaphragm, pericardium, chest wall, lung, thoracic vertebrae/spinal cord, as well as ipsilateral mediastinal lymph nodes. Distant recurrences were divided into lesions at the contralateral hemithorax (CHT), supraclavicular lymph nodes, abdomen, nonthoracic spinal cord/vertebrae, brain, or distant bone, or soft tissue. We further categorized recurrences as local, distant, or simultaneous local and distant. In addition, IHT and CHT recurrences to pleura, lung, and pericardium were combined for biomarker analysis.

### Biomarkers

Circulating SMRP and CA‐125 were routinely measured. For SMRP, the standard reference range was 0–1.5 nmol/L. For CA‐125, a reference range of 6–46 U/mL was used for premenopausal women, and 6–32 U/mL for men and postmenopausal women.

For patients with a recurrence, circulating biomarkers were collected within 60 days of three timepoints: (1) prior to surgery, (2) after surgery, and (3) at time of recurrence. An average level was calculated from those with multiple postoperative surveillance measurements without recurrence.

### Statistical analysis

Descriptive statistics were used to report demographic and clinical data, which are presented as the mean (±SD) or median (IQR) for continuous factors and frequencies for categorical variables. Mann–Whitney U tests (or Kruskal‐Wallis tests where appropriate) were used to verify the associations of biomarkers with clinical baseline variables and at recurrence. A Kruskal‐Wallis test was also used to evaluate the change of biomarker levels over time. The association between biomarkers, age and TV was quantified using the Spearman's correlation coefficient. Linear mixed‐effect, repeated‐measurement models were used to estimate the average change across timepoints of the biomarker levels. Poisson regression models were used to infer the (potentially) optimal biomarker cutoff to assess risk of recurrence after surgery. Overall survival (OS) was defined as the difference in time from the date of the surgery to the date of the most recent follow‐up or death from any cause. Disease‐free survival (DFS) was defined as the difference in time from surgery to either the first sign of recurrence or death. Alive and lost‐to‐follow‐up patients were censored at the time of their last visit. Multivariable Cox models adjusting for age, sex, TNM stage, intraoperative heated chemotherapy (IOHC), and TV were fitted after a preliminary run with univariable models. To account for a possible dependence of the biomarker level on the measurement timepoint (pre‐, postoperative, recurrence/surveillance) we included an interaction term of the corresponding biomarker (treated as a continuous variable) and the measurement time.

Tables S2 and S3 in the supplemental material show the corresponding hazard ratio (HR) estimates with corresponding 95% confidence intervals (CIs). All the statistical analyses were performed using the R statistical software,^23^ with a *p*‐value < 0.05 considered statistically significant.

## RESULTS

### Study participants and baseline characteristics

**FIGURE 1:**
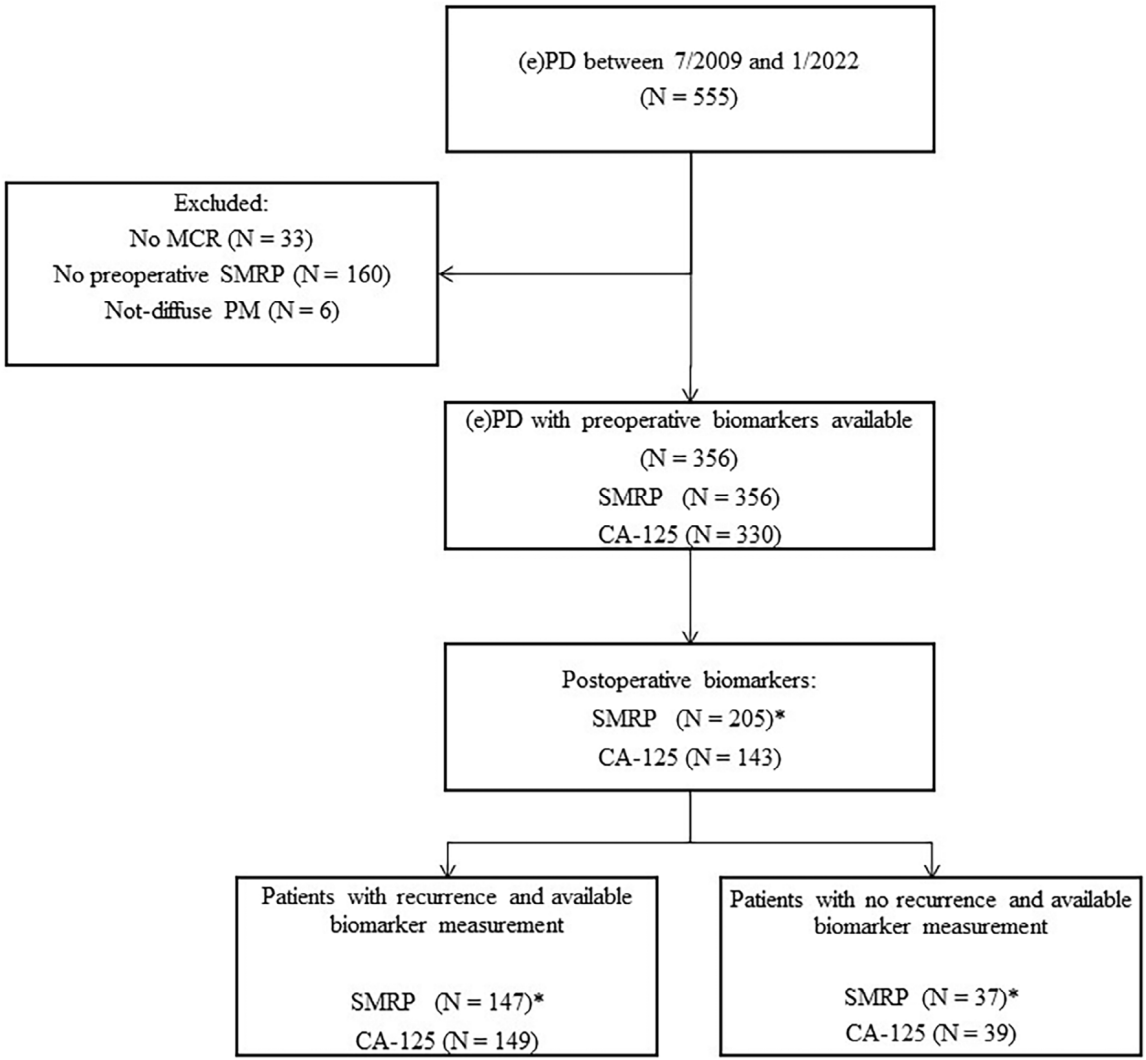
Study flow chart shows patient allocation and available biomarkers at different study timepoints. SMRP, soluble mesothelin‐related protein; 2. CA‐125, cancer antigen 125. *Represents only patients with epithelioid and biphasic subtypes.

Between September 2009 and January 2022, 555 PM patients underwent PD at our institution. Of these, 33 (8%) did not achieve MCR, 160 (28%) did not have preoperative SMRP available, and six (2%) had a diagnosis other than diffuse PM. Of the remaining 356 patients available for analysis, 276 (78%) had signs of recurrence by the end of follow‐up in January 2023. Figure 1 shows patient allocation and circulating biomarker availability at different study timepoints.

Table 1 presents the baseline characteristics of the study participants. The median age was 70 years, and most (267/356, 75%) patients were male. The histological subtype was epithelioid in 64% (228/356), biphasic in 31% (111/356), and sarcomatoid in 5% (17/356) of cases. Stage IB (169/356, 48%) was the most common TNM stage group. Most of the patients (302/356, 85%) received IOHC with 175 mg/m^2^ of cisplatin during surgery. Induction therapy was given for 25% (88/356) and adjuvant treatment for 58% (208/356) of patients.

**TABLE 1:** Patient and disease characteristics.

	Patients (*N* = 356)
Age, years, median (IQR)	70 (63–75)
Sex, male, n (%)	267 (75%)
Side, n (%)	
Right	220 (62%)
Left	135 (38%)
Bilateral	1 (0%)
Histological subtype, n (%)	
Epithelioid	228 (64%)
Biphasic	111 (31%)
Sarcomatoid	17 (5%)
Surgery type, n (%)	
Extended pleurectomy decortication^a^	300 (84%)
Pleurectomy decortication	56 (16%)
Extent of resection, n (%)	
Diaphragm	297 (83%)
Pericardium	182 (51%)
Pathological stage^b^	
Stage IA	26 (7%)
Stage IB	169 (48%)
Stage II	58 (16%)
Stage IIIA	63 (18%)
Stage IIIB	38 (11%)
Stage IV	2 (1%)
Tumor volume, cm^3^, median (IQR)^b^, ^c^	210 (63–462)
Clinical stage	
Stage I	97 (27%)
Stage II	78 (22%)
Stage III	111 (31%)
Stage IV	18 (5%)
IOHC, yes, n (%)	302 (85%)
Induction therapy, yes, n (%)	88 (25%)
Chemotherapy	66 (19%)
FAK‐inhibitor	21 (6%)
Immunotherapy	2 (1%)
Adjuvant therapy, yes, n (%)	208 (58%)
Chemotherapy	200 (56%)
Immunotherapy	4 (1%)
Radiotherapy	19 (5%)

Abbreviations: FAK, focal adhesion kinase; IQR, interquartile range; IOHC, intraoperative heated chemotherapy.

^a^ Extended PD includes resection of diaphragm or pericardium.

^b^ Tumor, node, metastasis American Joint Committee on Cancer eighth edition.

^c^ Available in 304 (85%) of cases.

### Preoperative biomarkers and clinical variables

**FIGURE 2:**
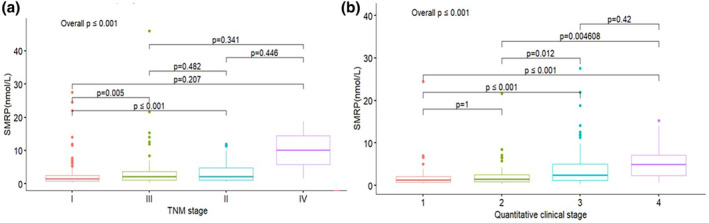
Box plot graph shows association with preoperative soluble mesothelin‐related protein (SMRP) and TNM stage (a) and clinical stage (b).

The mean (±SD) preoperative SMRP level was 2.9 ± 4.2 nmol/L, with a positive rate of 50% (177/356) (Table 2). Patients with epithelioid histology had higher preoperative SMRP levels compared to biphasic and sarcomatoid subtypes (*p* < 0.001). The preoperative SMRP levels were elevated in patients with advanced TNM (Figure 2, *p* < 0.001) and clinical stage groups (Figure 2, *p* < 0.001). Similarly, preoperative SMRP was significantly associated with TV (Spearman's correlation: 0.306; *p* < 0.001). No associations were observed between preoperative SMRP and age (*p* = 0.519), or sex (*p* = 0.733). When focused only on patients with positive preoperative SMRP levels, the association with histology (*p* < 0.001), TNM stage (*p* = 0.026), and TV (R = 0.446, *p* < 0.001) remained significant.

**TABLE 2:** Circulating tumor marker levels in different histological subtypes across study timepoints.

Tumor markers	Total		Epithelioid		Biphasic		Sarcomatoid	
	Mean ± SD	Positive rate^a^, %	Mean ± SD	Positive rate^a^, %	Mean ± SD	Positive rate^a^, %	Mean ± SD	Positive rate^a^, %
Preoperative								
SMRP, nmol/L	2.9 ± 4.2	50%	3.4 ± 4.6	58%	2.1 ± 3.4	38%	0.9 ± 0.4	12%
CA‐125, U/mL	51.2 ± 175.4	22%	55.2 ± 206.8	21%	48.4 ± 104.5	23%	16.0 ± 18.8	19%
Postoperative								
SMRP, nmol/L	0.9 ± 1.6^c^	9%^c^	1.0 ± 1.9	11%	0.7 ± 0.4	5%	‐	‐
CA‐125, U/mL	31.5 ± 89.9	22%	35.1 ± 111.3	23%	27.1 ± 21.2	24%	15.0 ± 9.3	11%
Recurrence								
SMRP, nmol/L	2.1 ± 3.3^c^	40%^c^	2.5 ± 4.0	40%	1.3 ± 1.0	30%	‐	‐
CA‐125, U/mL	66.0 ± 366.6	20%	81.5 ± 463.9	19%	47.1 ± 115.6	23%	7.4 ± 3.5	0%
Average surveillance^b^								
SMRP, nmol/L	1.0 ± 0.6^c^	19%^c^	1.0 ± 0.7	24%	0.9 ± 0.2	0%	‐	‐
CA‐125, U/mL	16.3 ± 11.1	5%	17.2 ± 12.3	7%	12.8 ± 5.4	0%	17.0 ± 9.9	0%

Abbreviations: CA‐125, cancer antigen 125; SMRP, soluble mesothelin‐related protein.

^a^ SMRP: Over 1.5 nmol/L; CA‐125 over 45 U/mL for premenopausal women, and over 32 U/ml for men and postmenopausal women.

^b^ Patients with no evidence of recurrence.

^c^ Represents only patients with epithelioid and biphasic subtypes (patients with sarcomatoid tumors were excluded from postoperative and recurrence analysis due to lack of SMRP shed in the bloodstream).

Preoperative CA‐125 was available for 93% (330/356) of patients with a mean (±SD) level of 51.2 **±** 175.4 U/mL and a positive rate of 22% (71/330). Spearman's correlation coefficient (for association with continuous variables), or Kruskal‐Wallis test (for association with categorical variables) showed no significant associations between preoperative CA‐125 and age (*p* = 0.751), sex (*p* = 0.477), histology (*p* = 0.898), TNM stage (*p* = 0.261), clinical stage (*p* = 0.096), or TV (*p* = 0.124).

### Circulating biomarkers at recurrence

Table S1 shows the distributions of anatomic recurrence sites. A total of 276 patients (76%) had a total of 1032 sites of recurrence. Most of the patients had local recurrence (253/356, 92%), while 137 (50%) had only local, 23 (8%) only distant, and 116 (42%) had both local and distant recurrences.

Due to lack of SMRP shed in the bloodstream by sarcomatoid tumors, the SMRP cohort was restricted only to patients with epithelioid and biphasic histology for the upcoming analysis. Of 147 patients with SMRP available at recurrence, 59 (40%) had SMRP levels above positive threshold with a mean (±SD) level of 2.1 ± 3.3 nmol/L. Patients with only distant recurrence (0.9 nmol/L ± 0.5) had lower levels than those with only local (1.8 ± 3.5 nmol/L) or both local and distant (2.5 ± 3.2 nmol/L; *p* = 0.011). Particularly, with respect to nonpleural and nonlymphatic recurrences, mean levels were higher in patients with pleural (2.3 ± 3.6 vs. 1.2 ± 1.0 nmol/L; *p* = 0.021) and lymphatic (2.1 ± 2.3 vs. 2.0 ± 4.2 nmol/L; *p* = 0.042) recurrences.

CA‐125 levels were available in 149 patients at recurrence, with a mean (±SD) of 66.0 ± 366.6 U/mL (Table 2). In contrast to SMRP, mean CA‐125 values were lower in those with only local recurrence (22.0 ± 50.6 U/mL) compared to distant only (87.3 ± 218.1 U/mL) and both distant and local recurrences (114.6 ± 552.8 U/mL; *p* < 0.001). Like SMRP, patients with metastasis to the lymphatic system had higher values (89.6 ± 482.5 U/mL) than those who did not (36.3 ± 103.7 U/mL; *p* = 0.004). Patients with abdominal recurrences had over six times higher mean values than those who did not (143.1 ± 607.3 U/mL vs. 23.4 ± 48.8; *p* < 0.001).

### Circulating biomarkers and surveillance

**FIGURE 3:**
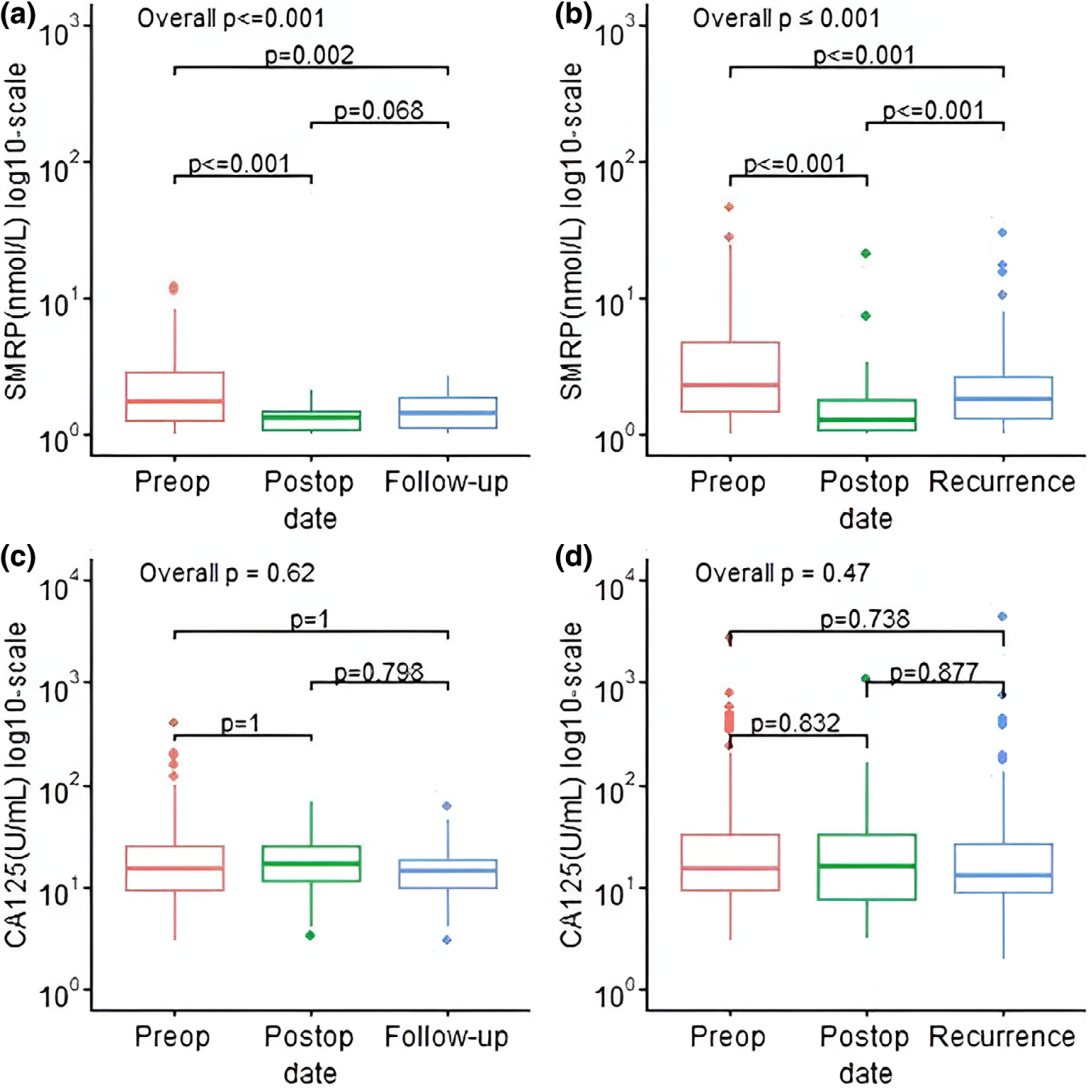
Change of biomarker levels in patients who had all timepoints available across study timepoints. Upper panel shows soluble mesothelin‐related protein (SMRP) on patients without (a) and with (b) a recurrence. The lower panel shows CA‐125 in patients who did not recur (c), and those who had a recurrence (d).

When analyzing only patients with biomarker levels available at all three timepoints (*N* = 113 for SMRP and *N* = 83 for CA‐125), 102 patients (90%) experienced a decline of SMRP after cytoreductive surgery with an average decline of 1.93 nmol/L (*p* < 0.001). After postoperative follow‐up, 87 patients (77%) with SMRP levels available experienced tumor recurrence. SMRP levels elevated an average of 0.79 nmol/L (*p* < 0.001) from post‐operation to recurrence (Figure 3, *p* < 0.001), with levels not significantly changed in patients without recurrence (Figure 3, *p* = 0.408). When selecting patients that had positive preoperative SMRP, the average postoperative levels decreased by 3.45 nmol/L (*p* < 0.001) and increased by 1.25 nmol/L from the post‐operation to recurrence (Figure SS1, *p* < 0.001).

There were no significant changes in levels of CA‐125 across the study points, regardless of the recurrence status (Figure 3, *p* = 0.51 for patients who recurred, and *p* = 0.62 for patients without recurrence). For patients with positive preoperative CA‐125 levels, there was a significant postoperative decrease of CA‐125 (Figure S1, *p* < 0.001). However, the postoperative surveillance levels remained unchanged in patients with (*p* = 1.00) and without (*p* = 1.00) recurrence.

### Circulating biomarkers and recurrence risk

Postoperative increase of SMRP levels by at least 0.7 nmol/L had an elevated recurrence risk (RR) of 1.40 (95% CI: 1.19–1.64, *p* < 0.001) for recurrence. A trend towards increased risk was seen in patients with positive preoperative SMRP values with RR of 1.37 (95% 0.98–1.89, *p* = 0.058) when surveillance SMRP increased by at least 0.45 nmol/L from postoperative levels. The association of CA‐125 levels and risk of recurrence was nonsignificant (*p* = 0.161).

### Circulating biomarkers and prognosis

After median follow‐up time of 70.4 months (95% CI: 60.2–81.6), 268 (75%) patients were deceased. The median OS for the study population was 25.6 months (95% CI: 21.1–29.5) and a median DFS was 12.1 months (95% CI: 10.8–13.8). After multivariable adjustments, neither preoperative, postoperative, or recurrence biomarkers were associated with OS (Table S2) or DFS (Table S3).

## DISCUSSION

Postoperative surveillance in PM is primarily based on longitudinal follow‐up with CT scans. The determination of recurrence can be challenging due to the complex morphology of the tumor, difficulty in achieving microscopic control, and overlap of evolving post‐surgical changes with inflammatory response and tumor recurrence both on anatomical and functional imaging. The addition of blood‐based biomarkers could act as an adjunct to radiological surveillance, and could lead to earlier and more accurate detection, diagnosis, and treatment of tumor recurrence. We have shown that the changes of SMRP follow closely the radiographic evidence of local recurrence, whereas changes in CA‐125 indicate presence of lymphatic or abdominal recurrence.

SMRP has been widely studied as a marker for diagnosis,^16^, ^17^ prognosis,^24^ and monitoring treatment response for PM.^8^ Nearly half of our patients had elevated SMRP levels preoperatively. As in previous studies, we demonstrated that SMRP values are more likely to be elevated in epithelioid PM.^8^, ^25^ In addition, we also observed higher SMRP levels in patients with high TV and advanced tumor stage. Conversely, preoperative CA‐125 levels were elevated only in one‐fifth of the patients with no clear associations with clinical characteristics. Previously, Creaney et al.^26^ compared the diagnostic ability of pretreatment circulating CA‐125 and SMRP in 117 PM patients. Serum CA‐125 levels were elevated in 42% of PM patients, but the addition of CA‐125 did not improve the diagnostic accuracy of SMRP. Similar to our findings, they observed no differences of CA‐125 levels across the histological subtypes in PM patients.

To date, no other studies have evaluated the association of tumor markers and anatomic site of recurrence in PM. Similar to extrapleural pneumonectomy (EPP).^27^, ^28^ we demonstrated that most of the recurrences occurred locally after PD. Mean SMRP levels were elevated in patients who had local recurrences, regardless of whether they had simultaneous distant recurrences. Specifically, patients with recurrences in the pleural space expressed elevated mean SMRP levels. In contrast to SMRP, mean CA‐125 levels were highest in patients with distant disease, especially those with lymphatic or abdominal spread. In a study of 60 peritoneal mesothelioma patients, the percentage of elevated preoperative CA‐125 was up to 65% in treatment naïve patients.^14^ They also demonstrated that CA‐125 levels correlated with the extent of peritoneal involvement and radiological presence of the disease after surgery. Another study confirmed that the CA‐125 levels were over twice as elevated in peritoneal mesothelioma patients compared to healthy controls.^29^

As previously discussed, epithelial tumors seem to be responsible for most of the SMRP production. Thus, surveillance of SMRP was limited to patients with nonsarcomatoid histology. First, we demonstrated that mean SMRP levels were decreased following resection. Subsequently, the average SMRP levels increased at recurrence but remained unchanged in patients without recurrence. Naturally, changes were more prominent in patients with positive preoperative values. This trend suggests that longitudinal SMRP surveillance could be useful in clinical postoperative care. Moreover, if the SMRP levels increased at least 0.7 nmol/L from postoperative value, there was an 40% elevation in the risk of recurrence. Most of the previous studies evaluating SMRP surveillance have studied patients receiving palliative systemic therapy. Some of these studies have included small number of surgically treated patients, where they have shown a trend towards substantial postoperative decrease of SMRP after surgery.^8^, ^30^, ^31^ Burt et al.^25^ reported similar results on SMRP monitoring in a subset of 66 patients with epithelioid histology that achieved MCR. The mean postoperative value at 3 months postoperatively decreased significantly in patients with a preoperative value >1.0 nmol/L. Additionally, an increase in SMRP levels was associated with recurrence: The rise of SMRP more than 48% from postoperative value had a 90% sensitivity and 93% specificity for recurrence. Nakamura and colleagues^32^ presented similar findings on 152 patients who underwent cytoreductive surgery after induction chemotherapy. They observed positive preoperative SMRP levels in 27% of patients with levels remaining positive in 4% of patients postoperatively. SMRP remained low in those without recurrence, while it became positive in 40% of patients with recurrence. These results, along with our findings, indicates that SMRP can be useful in the postoperative surveillance, especially in patients with elevated SMRP prior to MCR.

The efficacy of CA‐125 surveillance has not been previously studied in a surgical series of PM. Similar to SMRP, we found that the mean values decreased postoperatively, but the average decrease was statistically significant only in patients who had positive preoperative values. Moreover, there were no significant changes in surveillance levels after surgery, regardless of the recurrence status. Baratti et al.^14^ studied circulating CA‐125 in 60 peritoneal mesothelioma patients who underwent cytoreductive surgery. Among patients with elevated baseline levels, sensitivity of CA‐125 in diagnosing residual disease after surgery was 100% with specificity of 96%. Thus, it seems that CA‐125 could be of value in peritoneal mesothelioma patients, or in those with initial pleural disease with spread to the abdominal cavity.

We observed that none of the biomarker levels were associated with recurrence or survival. Previous studies of advanced PM patients have reported mixed results regarding the prognostic value of SMRP.^8^, ^17^, ^30^, ^33^ Prognostic significance of preoperative SMRP have been evaluated in two surgical series.^25^, ^34^ No clear associations with preoperative SMRP and survival were found in a cohort of 90 PM patients who underwent cytoreductive surgery.^34^ Another study explored the prognostic value of preoperative SMRP in 66 patients with epithelioid PM.^25^ They observed that high SMRP was associated with shorter DFS, but not with OS. The prognostic value of CA‐125 has not been previously studied in PM patients undergoing MCR. Cheng et al.^19^ studied a cohort of 41 advanced unresectable mesothelioma patients, including 23 PM and 18 peritoneal mesothelioma patients. They observed that CA‐125, with a cutoff of 280 U/mL, remained an independent prognostic factor (HR 3.4, 95% CI: 1.3–9.2) after adjustments. These inconsistent results reported by the present study and others, suggest that these CA‐125 markers cannot reliably be used as prognostic markers following cytoreductive surgery.

Despite the prospective nature of most of the data and standardized biomarker collection in our clinic, some inconsistencies of biomarker availability were observed. Thus, potential selection bias could not be excluded. Our study was also limited by the small number of patients available for surveillance analysis. This was particularly true for evaluation of CA‐125. Further, we did not account for the effect of other comorbid condition on baseline levels of SMRP or CA‐125. Weber et al.^13^ demonstrated that serum concentrations of both SMRP and CA‐125 are influenced by various factors such as age, serum creatine concentration, previous asbestos exposure, and storage time of samples. However, these factors are not typically accounted for clinically suggesting that our cohort represents a real‐world population with no major exclusion factors.

In conclusion, elevated preoperative SMRP levels were commonly observed in patients with high tumor burden along with epithelioid and biphasic histology. Longitudinal changes in SMRP levels correspond with a radiographic presence of disease. SMRP surveillance may aid in the detection of otherwise radiologically subtle local recurrences. The addition of CA‐125 may be clinically helpful for the diagnosis of abdominal recurrences.

## AUTHOR CONTRIBUTIONS

All authors had full access to the data in the study and take responsibility for the integrity of the data and the accuracy of the data analysis. Conceptualization: Raphael Bueno. Methodology: Juuso Paajanen, Raphael Bueno, John Kuckelman and William G. Richards. Investigation: Juuso Paajanen, Ahmed Sadek and William G. Richards. Formal analysis: Yue Xie, Emanuele Mazzola. Resources: Raphael Bueno. Writing—original draft: Juuso Paajanen. Writing—review and editing: All authors. Visualization: Ritu R. Gill. Supervision: Raphael Bueno. Funding acquisition: Raphael Bueno.

Raphael Bueno is guarantor of the content of the manuscript, including the data and analysis. Juuso Paajanen, Raphael Bueno, John Kuckelman and William G. Richards conceived and designed the study. Yue Xie and Emanuele Mazzola performed the statistical analysis, Juuso Paajanen, Ahmed Sadek and Kristina Sidopoulos collected the data and Ritu R. Gill evaluated the CT images.

## CONFLICT OF INTEREST STATEMENT

The authors disclose no potential conflicts of interest.

## Supporting information


**Figure S1.** Change of biomarker levels in patients who had positive preoperative values and all timepoints available across study timepoints. The upper panel shows SMRP on patients without (a) and with (b) a recurrence. The lower panel shows CA‐125 in patients who did not recur (c), and those who had a recurrence (d).
