# Supplementary material for: Circulating SMRP and CA‐125 before and after pleurectomy decortication for pleural mesothelioma
Source: Thorac Cancer. 2024 Apr 16;15(15):1237–45. doi: 10.1111/1759-7714.15264 (PMC11128371; doi:10.1111/1759-7714.15264)
Supplement: Supplementary file 1 — Figure S1. Change of biomarker levels in patients who had positive preoperative values and all timepoints available across study timepoints. The upper panel shows SMRP on patients without (a) and with (b) a recurrence. The lower panel shows CA‐125 in patients who did not recur (c), and those who had a recurrence (d). [file TCA-15-1237-s001.docx]

**Supplementary Table 1**. Initial tumor sites among 276 patients with recurrence.

| **Recurrence** | **N (% of all patients)** |
| --- | --- |
| **Local**  Chest wall  Pleura  Pericardium  Diaphragm  Lung  Spinal cord/vertebrae (thoracic)  Mediastinal lymph nodes/soft tissue mass  Intrathoracic lymph nodes | 253 (92%)  157 (57%)  212 (76%)  42 (15%)  65 (24%)  71 (26%)  11 (4%)  136 (49%)  48 (17%) |
| **Distant**  Supraclavicular lymph nodes  Contralateral chest  Pleura  Lung  Pericardium  Abdomen  Abdominal lymph nodes  Ascites  Liver  Abdominal wall  Peritoneum, Mesentery, Omentum  Retroperitoneum  Spleen  Renal  Adrenal glands  Pancreas | 139 (50%)  19 (7%)  21 (8%)  28 (10%)  2 (1%)  42 (15%)  27 (10%)  36 (13%)  20 (7%)  44 (16%)  4 (1%)  4 (1%)  5 (2%)  7 (3%)  3 (1%) |
| Distant Musculoskeletal  Bone  Subcutaneous  Soft tissue  Central nervous system  Brain  Spinal cord/vertebrae (non-thoracic) | 8 (3%)  3 (1%)  7 (3%)  2 (1%)  8 (3%) |

**Supplementary Table 2**. Results of multivariable Cox model for factors associating with overall survival.

|  | **SMRP**^†^ |  | **CA-125**^‡^ | |
| --- | --- | --- | --- | --- |
|  | **HR**^§^ **(95% CI**^¶^**)** | **P-Value** | **HR (95% CI)** | **P-Value** |
| **Sex, male** | 1.23 (0.98 – 1.55) | 0.073 | 1.29 (1.02 – 1.65) | 0.036 |
| **Age (continuous)** | 1.02 (1.01 – 1.04) | <0.001 | 1.04 (1.02 – 1.05) | <0.001 |
| **Tumor volume (continuous)** | 1.00 (1.00 – 1.00) | <0.001 | 1.00 (1.00 – 1.00) | <0.001 |
| **Histology**  Biphasic  Epithelioid  Sarcomatoid | (baseline)  0.62 (0.51 – 0.75)  - | <0.001  - | (baseline)  0.61 (0.41 – 0.72)  0.99 (0.64 – 1.55) | <0.001  0.985 |
| **Intraoperative chemotherapy,yes** | 0.55 (0.42 – 0.72) | <0.001 | 0.55 (0.41 – 0.72) | <0.001 |
| **TNM Stage**  Stage I  Stage II  Stage III+IV | (baseline)  1.62 (1.25 – 2.11)  1.68 (1.36 – 2.07) | <0.001  <0.001 | (baseline)  1.68 (1.27 – 2.22)  1.59 (1.28 – 1.98) | <0.001  <0.001 |
| **Biomarker level (continuous)** | 1.00 (0.98 – 1.03) | 0.859 | 1.00 (1.00 -1.00) | 0.398 |
| **Measurement time**  Preoperative  Postoperative  Recurrence/surveillance | (baseline)  0.91 (0.71 – 1.16)  1.07 (0.83 – 1.38) | 0.434  0.602 | (baseline)  1.00 (0.78 – 1.29)  1.04 (0.83 – 1.30) | 0.976  0.722 |
| **Interaction terms** | | | | |
| **Additional HR for one-unit biomarker increase at different measurement times**  Preoperative  Postoperative  Recurrence/surveillance | (baseline)  1.07 (0.98 – 1.17)  0.98 (0.93 – 1.04) | -  0.132  0.534 | (baseline)  1.00 (0.99 – 1.00)  1.00 (0.99 – 1.00) | -  0.846  0.759 |

^†^SMRP, soluble mesothelin-related protein; ^‡^CA-125, cancer antigen 125; ^§^HR, hazard ratio; ^¶^CI, confidence interval.

**Supplementary Table** **3**. Results of multivariable Cox model for factors associating with disease-free survival.

|  | **SMRP**^†^ |  | **CA-125**^‡^ | |
| --- | --- | --- | --- | --- |
|  | **HR^c^ (95% CI**^¶^**)** | **P-Value** | **HR (95% CI)** | **P-Value** |
| **Sex, male** | 1.24 (0.99 – 1.53) | 0.051 | 1.22 (0.97 – 1.54) | 0.088 |
| **Age (continuous)** | 1.01 (1.00 – 1.02) | 0.019 | 1.02 (1.01 – 1.03) | 0.001 |
| **Tumor volume (continuous)** | 1.00 (1.00 – 1.00) | <0.001 | 1.00 (1.00 – 1.00) | <0.001 |
| **Histology**  Biphasic  Epithelioid  Sarcomatoid | (baseline)  0.60 (0.50 – 0.72)  - | <0.001  - | (baseline)  0.61 (0.41 – 0.72)  1.19 (0.75 – 2.00) | <0.001  0.429 |
| **Intraoperative chemotherapy,yes** | 0.58 (0.46 – 0.76) | <0.001 | 0.54 (0.41 – 0.72) | <0.001 |
| **TNM Stage**  Stage I  Stage II  Stage III+IV | (baseline)  1.67 (1.32 – 2.12)  1.46 (1.21 – 1.77) | <0.001  <0.001 | (baseline)  1.77 (1.37 – 2.30)  1.44 (1.18 – 1.77) | <0.001  <0.001 |
| **Biomarker level (continuous)** | 1.02 (1.00 – 1.05) | 0.051 | 1.00 (1.00 – 1.00) | 0.506 |
| **Measurement time**  Preoperative  Postoperative  Recurrence/surveillance | (baseline)  1.05 (0.94 – 1.32)  1.19 (0.94 – 1.50) | 0.658  0.151 | (baseline)  0.99 (0.78 – 1.25)  1.08 (0.87 – 1.33) | 0.931  0.485 |
| **Interaction terms** |  |  |  |  |
| **Additional HR for one-unit biomarker increase at different measurement times**  Preoperative  Postoperative  Recurrence/surveillance | (baseline)  1.05 (0.95 – 1.16)  0.96 (0.92 – 1.01) | 0.319  0.131 | (baseline)  1.00 (0.99 – 1.00)  1.00 (0.99 – 1.00) | 0.937  0.786 |

^†^SMRP, soluble mesothelin-related protein; ^‡^CA-125, cancer antigen 125; ^§^HR, hazard ratio; ^¶^CI, confidence interval

**Supplementary Figure 1.**


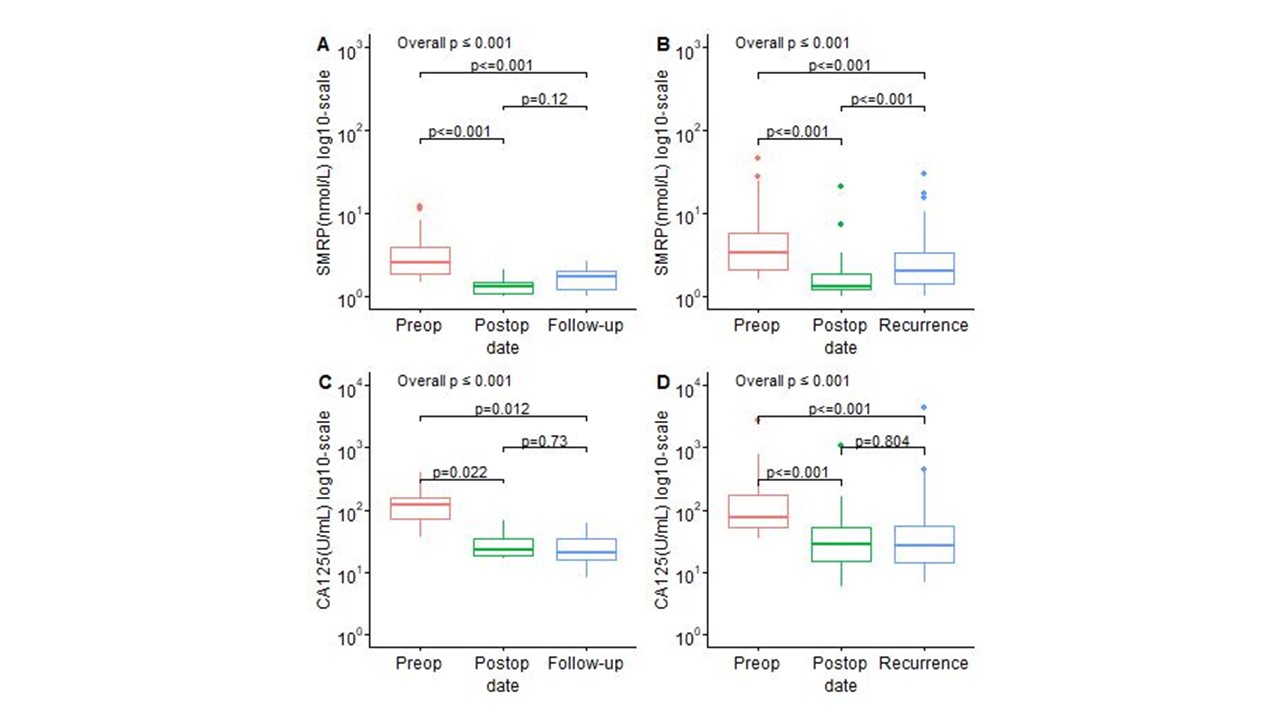


**Supplementary Figure 1.** Change of biomarker levels in patients who had positive preoperative values and all timepoints available across study timepoints. Upper panel shows SMRP on patients without (A) and with (B) a recurrence. The lower panel shows CA-125 in patients who did not recur (C), and those who had a recurrence (D).
